# Case Report: Clinical and Pathological Findings of a Recurrent C3 Glomerulopathy With Superimposed Membranoproliferative Glomerulonephritis Pattern and Cryoglobulinemia Associated With COVID-19

**DOI:** 10.3389/fped.2022.827466

**Published:** 2022-03-04

**Authors:** Nastaran Daneshgar, Peir-In Liang, Christina J. Michels, Carla M. Nester, Lyndsay A. Harshman, Dao-Fu Dai

**Affiliations:** ^1^Department of Pathology, Carver College of Medicine, University of Iowa, Iowa City, IA, United States; ^2^Department of Pathology, Kaohsiung Medical University Hospital, Kaohsiung Medical University, Kaohsiung, Taiwan; ^3^Division of Pediatric Nephrology, University of Iowa Stead Family Department of Pediatrics, University of Iowa, Iowa City, IA, United States; ^4^Molecular Otolaryngology and Renal Research Laboratories, Carver College of Medicine, University of Iowa, Iowa City, IA, United States

**Keywords:** COVID-19, glomerulonephropathy, cryoglobulinemia, case report, kidney transplant, membranoproliferative glomerulonephritis (MPGN), C3 glomerulopathy (C3G)

## Abstract

Coronavirus disease 2019 (COVID-19) may cause a wide spectrum of kidney pathologies. The impact of COVID-19 is unclear in the context of the complement system abnormalities, including C3 glomerulopathy (C3G). In this report, we describe a young adult receiving a kidney transplant for C3 glomerulopathy (C3G), a disorder of the alternative complement pathway. The patient developed a recurrent C3G ~7 months after transplantation. His post-transplant course was complicated by SARS-CoV-2 infection. There was a progression of glomerulonephritis, characterized by *de novo* immune-complex mediated membranoproliferative glomerulonephritis pattern of injury with crescentic and necrotizing features, along with positive immunoglobulins, persistent IgM staining and the presence of cryoglobulinemia. COVID-19 may have aggravated the inherent complement dysregulation and contributed to cryoglobulinemia observed in this patient. Our study of 5 sequential kidney allograft biopsy series implicates that COVID-19 in this patient promoted a superimposed immune complex-mediated glomerulonephritis with membranoproliferative glomerulonephritis (MPGN) pattern and cryoglobulinemia, which was a potentiating factor in allograft loss. This work represents the first report of cryoglobulinemic GN after COVID-19.

## Introduction

Coronavirus disease 2019 (COVID-19) may lead to exaggerated activation of the complement system (1). The impact of COVID-19 is unclear among patients with abnormalities of the complement system, including C3 glomerulopathy (C3G). C3G is a rare disorder of the alternative complement pathway associated with progression to end-stage kidney disease (ESKD) and a high risk for disease recurrence following kidney transplantation. We report the clinicopathological correlates for a kidney transplant recipient with a history of C3G who experienced COVID-19 amid post-transplant C3G recurrence.

## Clinicopathological Correlation

**Figure 1:**
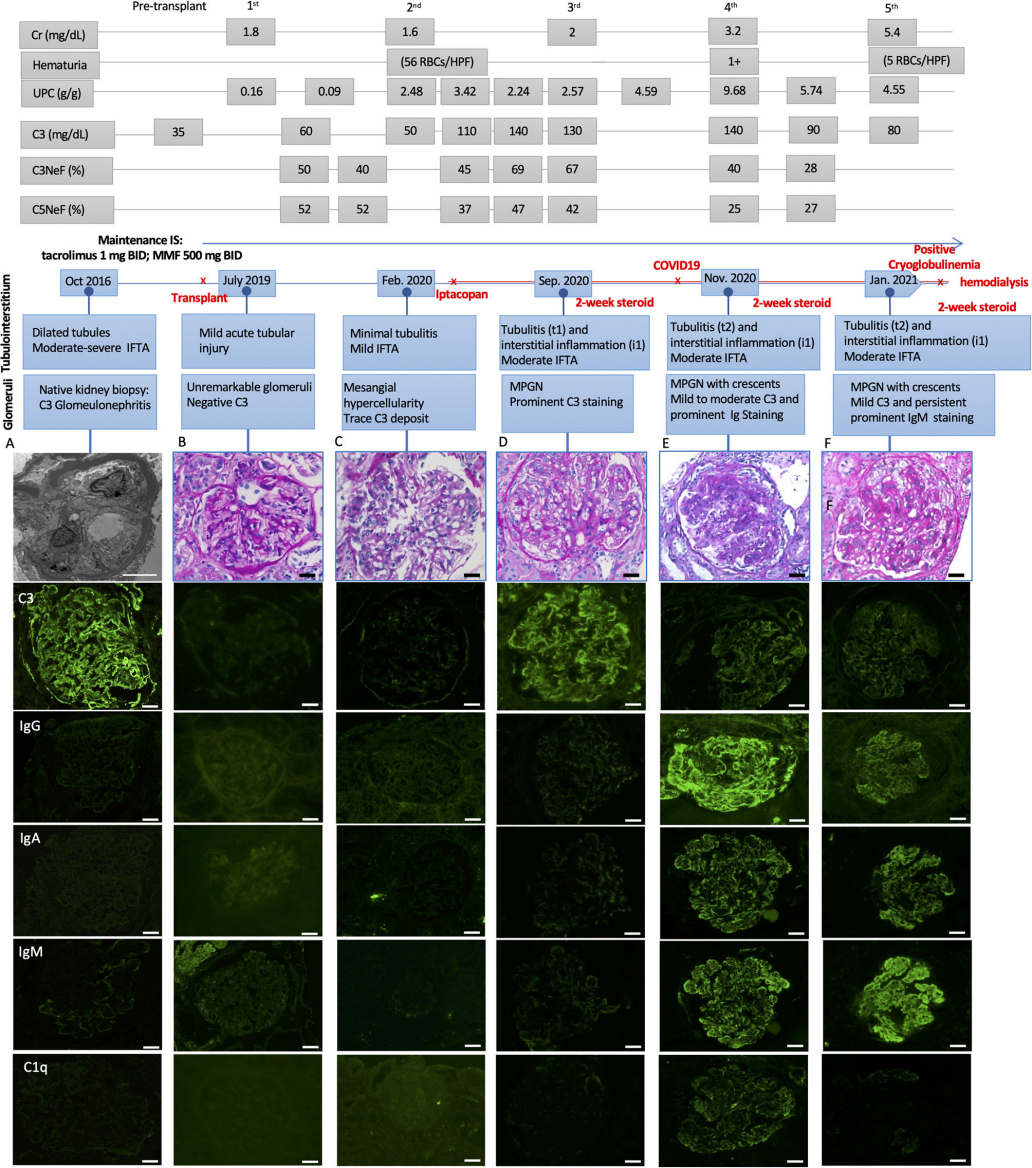
Light microscopic and immunofluorescence findings in a patient with C3G recurrence and COVID-19 infection with respective laboratory results, including serum creatinine (Cr), hematuria, urine protein creatinine ratio (UPC), C3, C3, and C5 Nephritic factors (Normal ranges: C3 > 85 mg/dL, C3Nef < 20%, C5Nef < 20%). **(A)** The native kidney biopsy showing intramembranous band-like electron dense deposition under electron microscope with prominent C3 staining (3+) and negative immunoglobulins staining. **(B)** The first biopsy 4 weeks after deceased-donor renal transplantation showing unremarkable glomeruli with negative immunofluorescence staining and mild acute tubular injury. **(C)** The second biopsy showing mild mesangial and focal endocapillary hypercellularity with mild glomerular C3 staining. **(D)** The third biopsy showing focal membranoproliferative glomerulitis (MPGN) lesion with prominent C3 staining (3+) and negative immunoglobulins staining. There was mild tubulointerstitial inflammation, suggestive of Banff borderline T-cell mediated rejection, for which 2-week steroid was given. **(E)** The fourth biopsy showing MPGN pattern with focal crescentic lesions, mild to moderate C3 staining (1–2+), prominent immunoglobulins staining, including IgG (3+), IgA (2+) and IgM (3+), and mild C1q staining (trace-1+). There was mild to moderate tubulointerstitial inflammation, suggestive of Banff borderline T-cell mediated rejection, for which 2-week steroid was given. **(F)** The fifth biopsy showing MPGN pattern with diffuse crescentic lesions, mild C3 staining (1+) and persistent prominent IgM staining (3+), mild to moderate IgG and IgA (1–2+), negative C1q staining. There was mild to moderate tubulointerstitial inflammation, suggestive of Banff borderline T-cell mediated rejection, for which another 2-week steroid was given. Scale bar: 50 μm, EM scale bar: 5 μm.

A 19-year-old male with ESKD secondary to C3G underwent a deceased donor kidney transplant. His pre-transplant history was notable for the presence of serum C3 nephritic factor, which led to his complement dysregulation and C3G. The native kidney biopsy taken 2 years before the transplant was characterized by diffuse proliferative and crescentic glomerulonephritis with prominent C3 (3+) and negative immunoglobulins staining. Electron microscopy (EM) showed ribbon-like dense transformation of the lamina densa of the glomerular basement membrane (GBM) consistent with a dense deposit disease (DDD) (Figure 1A).

**Figure 2:**
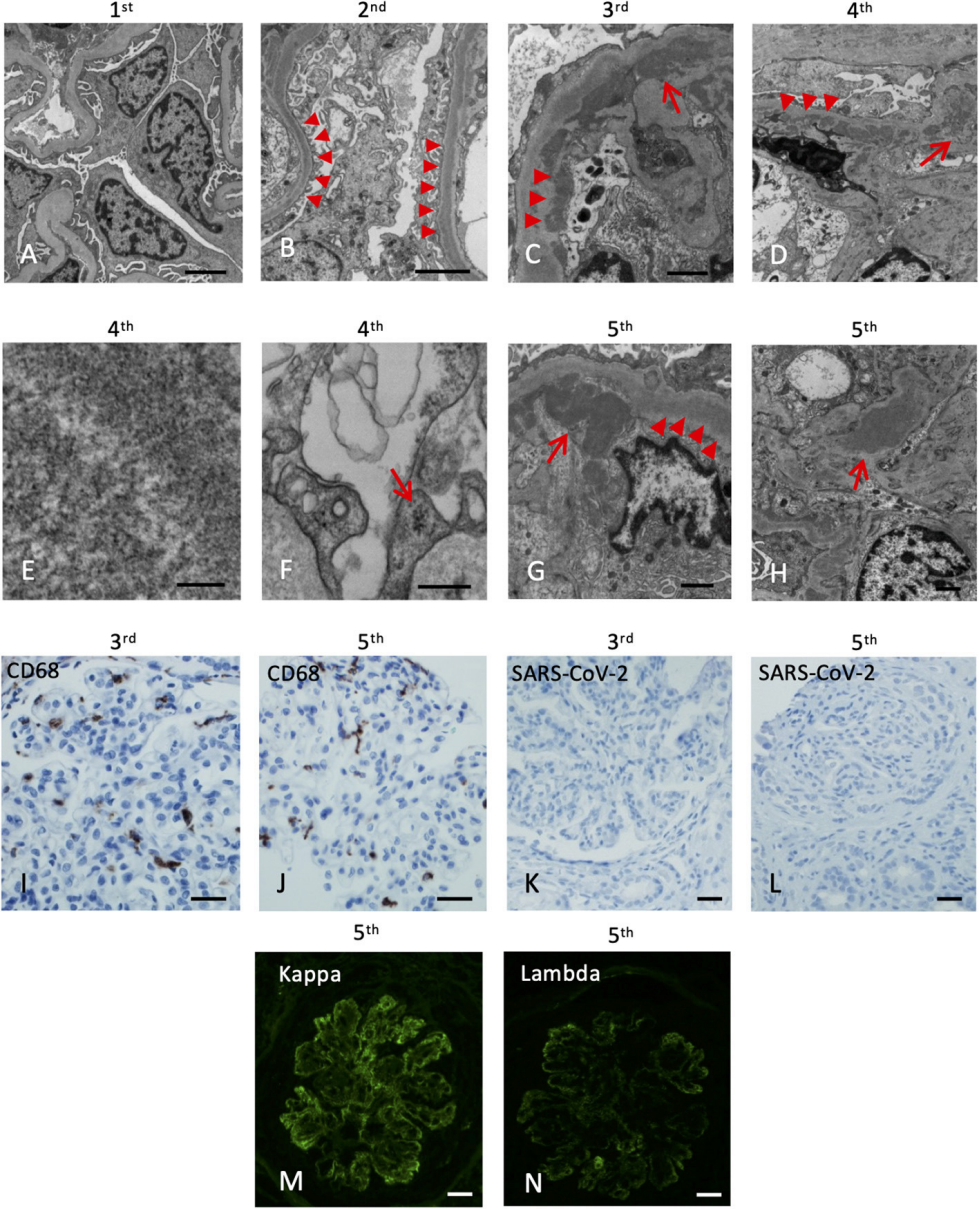
Electron microscopy and immunohistochemistry of the five kidney allograft biopsies. The labels represent the sequence of the biopsies. **(A)** Electron microscopy of the 1st renal biopsy showing no electron-dense deposition (Scale bar: 2 um). **(B)** Electron microscopy of the 2nd renal biopsy showing intramembranous electron-dense deposits (arrowheads, Scale bar: 2 um). **(C)** Subendothelial (arrowheads) and mesangial (arrow) electron-dense deposits (Scale bar: 1 um). **(D)** Abundant depositions in the intramembranous (arrowheads) and the paramesangial area (arrow) of the 4th renal biopsy (arrow) (Scale bar: 2 um). **(E)** High magnification revealed the deposits composed of vague, short, organized “wormy” fibrils substructure (Scale bar: 100 nm). **(F)** Tubuloreticular inclusions in the endothelium (Scale bar: 300 nm). **(G)** Abundant depositions in the intramembranous (arrowheads) and the subendothelial areas (arrow) of the 5th renal biopsy (Scale bar: 1 um). **(H)** Mesangial depositions in the 5th renal biopsy (arrow). (Scale bar: 1 um). **(I,J)** The CD68 immunostaining of the 3rd and 5th renal biopsy revealed increase histiocytic infiltrates in the glomeruli. **(K,L)** Immunohistochemical staining of SARS-CoV-2 nucleocapsid protein of the 3rd and 5th renal biopsy are both negative (Scale bar: 50 um). **(M,N)** Immunofluorescence of the 5th biopsy shows positive Kappa (3+) and Lambda (2+) staining (Scale bar: 50 um).

His post-transplant course was uneventful, with a nadir serum creatinine of 1.3 mg/dL within 2 weeks of transplant. Maintenance immunosuppressants included tacrolimus 1 mg BID with a therapeutic goal of 3–5 ng/mL and mycophenolate (MMF) at 500 mg BID. Due to elevated serum creatinine (1.8 mg/dL), a kidney biopsy (#1) was performed 1-month post-transplant which showed mild acute tubular injury as the only pathological finding. There was no evidence of rejection or disease recurrence (Figure 1B) with negative immunofluorescence (IF) studies and no evidence of deposits on EM (Figure 2A). Thus, maintenance immunosuppressants were continued without any adjustment. Approximately 7 months post-transplant, kidney biopsy (#2) was performed due to new-onset microscopic hematuria (56 RBCs/HPF) and sub-nephrotic proteinuria (2.48 by UPCR), which demonstrated early recurrent C3G characterized by mild mesangial and endocapillary hypercellularity; mild glomerular staining for C3 (1+) but negative for other Immunoglobulins (Figure 1C); and numerous intramembranous and mesangial electron-dense deposits on EM (Figure 2B). His serum C3 was decreased (50 mg/dL). The patient was started on a new complement inhibitor study drug, iptacopan the first dose at 10 mg BID (4/1/2020), with weekly dose escalation to 50 mg BID, 100 mg BID, then to full dose at 200 mg BID (4/21/20). This full dose was maintained for ~11 months. Iptacopan is a factor B inhibitor that blocks the activation of the alternative complement pathway. His renal function was stable after iptacopan treatment. At ~14 months post-transplant, he developed nephrotic range proteinuria, significant microscopic hematuria with a serum creatinine of 2 mg/dL. A kidney biopsy (#3) performed at that time demonstrated mesangial and endocapillary hypercellularity with accentuated lobulation and segmental duplication of the GBM (Figure 1D). Additional findings included mild tubulitis (t1) and lymphocytic infiltrates in the interstitium (i1), suggestive of Banff borderline acute cellular rejection (T-cell mediated rejection), for which he received 2-week steroids, including a pulse methylprednisolone (Figure 1D). Immunofluorescence studies showed prominent glomerular C3 staining (3+). EM showed electron-dense deposits in the subendothelial, mesangial, and intramembranous regions. Segmental GBM showed elongated intramembranous deposits in a ribbon-like distribution, although dark osmiophilic deposits, characteristics of DDD, were not identified. Rare duplication of GBM with cell interposition was noted (Figure 2C). The tubuloreticular inclusions were not identified. These glomerular findings were consistent with a recurrent C3G. Examination of donor specific antibodies (DSA) was negative, excluding the diagnosis of antibody mediated rejection.

Two months later (16 months post-transplant), the patient presented with worsening peripheral edema, hypoalbuminemia, hypertension, nephrotic range proteinuria, microscopic hematuria, and a rapid rise in serum creatinine level from 1.6 to 3.2 mg/dL. There was no fever, diarrhea, myalgia, respiratory symptoms, or loss of taste / smell. As COVID-19 testing is routinely performed during the pandemic, the patient was incidentally tested positive for COVID-19 by PCR upon admission. Kidney biopsy (#4) showed cellular crescents (2/13 glomeruli, ~15%) and fibrocellular crescents (5/13 glomeruli, ~23%). While the intensity of glomerular C3 staining decreased to 1–2+ (Figure 1E), there were new findings of glomerular IgG (3+), IgM (3+), IgA (2+), and C1q (trace) staining. EM showed numerous electron-dense deposits in the subendothelial, intramembranous, and mesangial areas. Some of these deposits had a repetitive pattern of vague, short, organized fibril substructures (Figures 2D,E). Tubuloreticular inclusions were now found in some endothelial cells (Figure 2F). These findings supported a “full-house” immune-complex mediated glomerulonephritis (IC-GN) with features of MPGN and crescents formation. There was also borderline acute T-cell mediated rejection by Banff criteria, for which he received another 2-week steroids, including a pulse methylprednisolone.

The patient's kidney function worsened (serum creatinine 4.48 mg/dL) with heavy proteinuria and hematuria (Figure 1). He subsequently developed low C4 (<5 mg/dL). His C3 remained slightly below normal at 80–85 mg/dL. A fifth kidney biopsy (#5) at 19 months post-transplant demonstrated diffuse fibrocellular (9/23 glomeruli, 39%) and fibrous crescentic lesions (6/23, 26%). The remaining glomeruli showed MPGN pattern (Figure 1F) with the progression of interstitial fibrosis and tubular atrophy (20–25%). There was acute tubular injury, moderate tubulitis (t2), and mild interstitial inflammation (i1). Immunofluorescence showed decreased staining intensity for IgG (3+ to 1+) and C3 (2+ to 1+) but persistent IgM staining (3+), with the pattern of granular, predominantly capillary walls staining, which was also positive for Kappa (3+) and Lambda (2+) (Figures 2M,N). EM showed electron-dense deposits in the intramembranous, mesangial, and subendothelial regions (Figures 2G,H).

Additional laboratory evaluations showed negative ANCA, myeloperoxidase and proteinase 3 antibodies, double-stranded DNA, and antinuclear antibodies (ANA) but positive cryoglobulins (5 mg/dL). He was not receiving any medications known to cause lupus-like nephritis. A thorough evaluation for other infectious etiologies, including viral hepatitis panels was negative. IHC studies for CD68, a marker of macrophages, highlighted infiltration of macrophages in the glomeruli in biopsy #3 (Figure 2I) and #5 (Figure 2J). IHC studies using anti-SARS-CoV-2 Nucleocapsid protein (performed on 3rd to 5th biopsies; no tissue left from 4th biopsy) were negative, and no viral-like particles were identified by EM (Figures 2K,L). Taken together, the last two biopsies demonstrated MPGN pattern of injury with several features suggestive of cryoglobulinemic glomerulonephritis. After ~22 months post-transplant, despite normalization of serum C3 and improvement in serum C3Nef and C5Nef, his allograft progressed to ESKD.

## Discussion

We described a kidney transplant patient with early recurrence of C3G, later superimposed with immune complex-mediated MPGN and cryoglobulinemia following SARS-CoV-2 infection. C3G is caused by dysregulation of the alternative complement pathway (2). The overall kidney prognosis is poor. Most C3G will progress to end-stage kidney disease (3), with a high risk for disease recurrence (~50–80%) causing allograft failure after transplantation (4).

Our patient developed C3G recurrence at ~7 months post-transplant. The normalization of serum C3 levels and the decrease in the intensity of glomerular C3 staining in the allograft after iptacopan suggests a gradual remission of the complement abnormalities. His clinical course was complicated by COVID-19 infection. Following COVID-19, he was noted to have hypocomplementemia with low C4 (<5 mg/dL) and borderline low C3 (83 mg/dL). The last two biopsies demonstrated an immune-complex mediated glomerulonephritis with MPGN and crescentic lesions, characterized by strong immunoglobulin M and immunoglobulin G staining. The persistent IgM staining (biopsy #5), presence of substructures within electron-dense deposits, and the progression of MPGN despite improvement of C3 argue against C3G as the main driver of worsened kidney function. Rather, these findings suggest a superimposed immune-complex mediated glomerulonephritis as the catalyst for deteriorating kidney function. Laboratory examination after COVID-19 infection showed positive cryoglobulinemia, concurrent with the deterioration of serum creatinine, progressive nephrotic range proteinuria and hematuria.

Kidney involvement has been reported in COVID-19, ranging from 17 to 56.9% of patients (5, 6). Common pathological findings in COVID-19 related nephropathy include acute tubular injury (7), collapsing glomerulopathy and thrombotic microangiopathy (8). Findings of crescentic glomerulonephritis, including ANCA-vasculitis and anti-GBM disease, and immune complex glomerulonephritis with full house immunofluorescence have been reported in rare cases of COVID-19 (8–10). The sensitivity and specificity of SARS-CoV-2 detection in kidney tissues are not well-established, with the controversial interpretation of “viral-like particles” identified by EM (11–13). In these post-COVID-19 kidney biopsies, IHC studies using anti-SARS-CoV-2 Nucleocapsid protein and attempts to identify viral particles by EM were both negative.

We hypothesize that COVID-19 associated cryoglobulinemia and membranoproliferative pattern of glomerulonephritis accelerated the progression of allograft dysfunction in this patient with early C3G recurrence. The repetitive substructures of electron-dense deposits in the GBM, subendothelial areas, and mesangium resembled the so-called “short wormy” substructure reported in cryoglobulinemic glomerulonephritis (14). Cryoglobulinemic GN is well-known to be associated with viral infections (such as hepatitis C or B virus), autoimmune diseases, and lymphoma (15, 16). All of these conditions (other than COVID-19) were absent in this patient. Tubuloreticular inclusions, a marker of increase in systemic interferons, were not present in biopsy #3 but were subsequently noted in biopsy #4 following COVID-19, which is well-known to associate with a cytokine surge (17). To the best of our knowledge, this is the first report of cryoglobulinemic GN after SARS-CoV-2 infection.

The potential impact of inherent complement dysregulation in the setting of COVID-19 infection cannot be understated. COVID-19 has been shown to activate complement pathways (1) which can further aggravate the underlying complement dysregulation of our patient. A previous study on a patient with relapsed atypical hemolytic uremic syndrome (HUS) after COVID-19 supports this notion (18). IC-GN in the setting of COVID-19 has been recently reported in a patient with HUS (10). Since a “full-house” IC-GN with diffuse electron-dense deposits in mesangium is rare in HUS, this feature was more likely associated with COVID-19(10). Our patient experienced early, recurrent C3G and later developed a superimposed IC-MPGN with features of cryoglobulinemic GN following SARS-CoV-2 infection (Figure 1) without clinical or pathological features of HUS, or antibody mediated rejection. Our report suggests that COVID-19 infection could be an additional, significant risk factor for allograft loss in transplant recipients with inherent complement dysregulation.

The strength of our study include a thorough follow up of the patient with 5 serial kidney biopsies and laboratory workups. However, given the complexity of his clinical course, proving that COVID-19 is the direct cause of immune complex-mediated glomerulonephritis with membranoproliferative glomerulonephritis (MPGN) pattern and cryoglobulinemia was not possible, although many other etiologies of cryoglobulinemia and MPGN were excluded.

## Patient Perspective and Consent

The patient provided verbal consent to have his story shared in this manner in hopes that it would improve care for others.

## Data Availability Statement

The original contributions presented in the study are included in the article/supplementary material, further inquiries can be directed to the corresponding authors.

## Ethics Statement

Written informed consent was obtained from the individual(s) for the publication of any potentially identifiable images or data included in this article.

## Author Contributions

ND, P-IL, LH, and D-FD performed renopathological study and manuscript writing. CN provided critical revision and editing of the manuscript. All authors contributed to the article and approved the submitted version.

## Funding

This work was supported by NIH K08 HL145138-02 (DFD).

## Conflict of Interest

The authors declare that the research was conducted in the absence of any commercial or financial relationships that could be construed as a potential conflict of interest.

## Publisher's Note

All claims expressed in this article are solely those of the authors and do not necessarily represent those of their affiliated organizations, or those of the publisher, the editors and the reviewers. Any product that may be evaluated in this article, or claim that may be made by its manufacturer, is not guaranteed or endorsed by the publisher.
